# Advancing vaccine technology through the manipulation of pathogenic and commensal bacteria

**DOI:** 10.1016/j.mtbio.2024.101349

**Published:** 2024-11-16

**Authors:** Khristine Kaith S. Lloren, Amal Senevirathne, John Hwa Lee

**Affiliations:** College of Veterinary Medicine, Jeonbuk National University, 79 Gobong-ro, Iksan City, Jeollabuk-do, 54596, Republic of Korea

**Keywords:** Bacterial engineering, Vaccine technology, Pathogenic bacteria, Commensal bacteria, Vaccination strategy, Vaccine delivery

## Abstract

Advancements in vaccine technology are increasingly focused on leveraging the unique properties of both pathogenic and commensal bacteria. This revolutionary approach harnesses the diverse immune modulatory mechanisms and bacterial biology inherent in different bacterial species enhancing vaccine efficacy and safety. Pathogenic bacteria, known for their ability to induce robust immune responses, are being studied for their potential to be engineered into safe, attenuated vectors that can target specific diseases with high precision. Concurrently, commensal bacteria, which coexist harmlessly with their hosts and contribute to immune system regulation, are also being explored as novel delivery systems and in microbiome-based therapy. These bacteria can modulate immune responses, offering a promising avenue for developing effective and personalized vaccines. Integrating the distinctive characteristics of pathogenic and commensal bacteria with advanced bacterial engineering techniques paves the way for innovative vaccine and therapeutic platforms that could address a wide range of infectious diseases and potentially non-infectious conditions. This holistic approach signifies a paradigm shift in vaccine development and immunotherapy, emphasizing the intricate interplay between the bacteria and the immune systems to achieve optimal immunological outcomes.

## Graphical abstract

**Figure:**
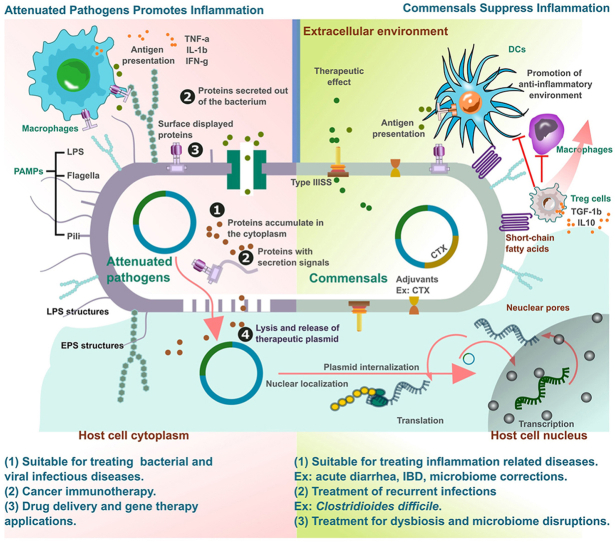

## 1 Introduction

Bacterial species have been instrumental in advancing vaccine technologies by providing innovative vaccine development and delivery methods. Since Louis Pasteur created the first-ever bacterial vaccine against chicken cholera in 1870, the exploitation of bacterial cells has come a long way, reaching today's highly advanced personalized vaccine technologies [1]. These minute entities are ubiquitously present all around us, on the surface of our skin and in gastrointestinal tracts serving as allies in digestion and strengthening the immunity against various infectious diseases [2]. Bacterial genomes range in size, offering high potential for genetic manipulation and could carry a significant payload of antigens making them unique among other strategies of vaccine delivery such as viral vectors, nanoparticle-based techniques, and lipid-based immunization.

In vaccine delivery technology, the active delivery method offered by bacterial vectors can be advantageous over the passive delivery like in lipid-based and nanoparticle-based strategies, particularly in the efficient systemic delivery of antigens. Bacterial outer membrane structures are highly complex and contain multiple immunological structures that can trigger broad immune responses and efficiently present antigens to antigen-presenting cells (APCs). Engineered bacteria can also be utilized in any immunization route, including parenteral and mucosal routes with remarkable efficacy. Key immunological properties such as active chemotaxis and DIVA (Differentiating Infected from Vaccinated Animals) capabilities are highly applicable in bacteria-mediated vaccine delivery platforms [3]. DIVA is a crucial aspect of vaccine development, especially in Veterinary Medicine, as this allows for differentiation between naturally infected animals and those that have been vaccinated. Compared to viral vectors, bacterial vectors are easily engineered for DIVA by including or excluding specific antigens or marker genes. For example, a bacterial vaccine may lack a non-essential antigen that is present in the wild-type pathogen, enabling differentiation through serological tests [4]. Other key advantages of the bacteria-based platform over other delivery platforms include genetic engineering flexibility, cost-effectiveness and stability.

Over the past few decades, the use of bacterial strains as an effective vaccine strategy has grown significantly. One of the most successful bacterial vaccine strains is the *Salmonella enterica* serovar Typhi Ty21a developed against typhoid fever which elicits robust immune responses, saving many lives, especially in endemic areas. *Listeria monocytogenes* strains have also emerged as promising vaccine carriers, especially against cancer immunotherapy. They can deliver tumor-associated antigens, which stimulate anti-cancer immunity against cancer types such as melanoma. Clinical trials have also provided evidence of enhanced cytotoxic T-cell responses by *Listeria* vaccine strains, highlighting their potential as anti-cancer vaccines. Another success story in harnessing bacterial cells is the development of the *Mycobacterium bovis* (BCG) vaccine against tuberculosis (TB) in the early 20th century, which was also found to be highly effective in preventing non-invasive bladder cancers. Furthermore, the HIV vaccine created using *Lactococcus lactis* and *Bordetella pertussis* intra-nasal vaccine candidates can also be recognized as remarkable achievements of bacterial vaccine technology [5].

Vaccine development faces numerous challenges, including lengthy screening timelines, rigorous selection of protective antigens, thorough preliminary investigations, strategic design of delivery methods, and the final deployment as an effective vaccine. The recent emergence of the COVID-19 pandemic highlighted the critical importance of rapid development and production of efficient vaccines to safeguard highly vulnerable populations [6]. Although other vaccine development platforms are generally safe, their long production times can leave many people vulnerable to infection and death. In such situations, bacteria-mediated vaccines can be explored, produced and scaled up relatively easily to meet the increased demand for efficient vaccines, especially during a pandemic. Many studies provide strong evidence that bacterial vaccine platforms can be successfully used against infectious diseases, immune modulation, and microbiome-based therapeutics [7,8]. Despite the tremendous potential of bacterial vaccines, safety-related issues remain a major concern. Bacteria can acquire and share harmful genetic elements like antibiotic-resistant genes and toxin-producing genes. However, by thoroughly examining the bacterial genomes and making precise genetic modifications, these issues can be effectively addressed. Combining biochemical and genetic techniques is often more effective for developing bacterial strains and ensuring their safe use as vaccines.

The development of vaccines and immunotherapies mediated by bacteria has marked several significant milestones throughout history, shaping the landscape of preventive and therapeutic medicine, from the early use of attenuated bacterial strains like BCG against tuberculosis [9] to the modern engineering of bacterial vectors that can be integrated with recombinant DNA technology [10] for the development of efficient vaccines until the current exploration on the essential role of bacteria in human microbiome in modulating immune responses for therapy [11]. Through various advanced engineering methods, there is a rise in the next generation of bacterial therapies for tailor-made personalized preventive and treatment applications [12].

Here, we review the unique biological properties of pathogenic and commensal bacteria, highlighting the current advances in genetic engineering techniques applied to these bacterial cells for safe and enhanced vaccine efficacy and delivery, the integration of cutting-edge delivery systems and the emerging trends in exploiting bacteria for personalized vaccine development. This will further expand our insights into how bacterial-based technologies could pave the way in addressing current and emerging global health challenges in a fast-changing environment that has expedited the emergence of novel variants of global pathogens.

### 1.1 The immunology of vaccines

Vaccination is one of the most significant public health achievements, playing a vital role in reducing the global burden of infectious diseases. Vaccines function by priming the immune system to identify and combat specific pathogens, effectively training the body to mount a defense without causing illness. This preparation enables the immune system to respond rapidly and effectively if exposed to the actual pathogen in the future, thereby preventing or significantly reducing the severity of the disease. They introduce carefully selected antigens, which can include inactivated pathogens, live attenuated organisms, or subunits like proteins or polysaccharides, into the host's immune system. These antigens are detected by pattern recognition receptors (PRRs) on immune cells, triggering an innate immune response that activates professional antigen-presenting cells (APCs), such as dendritic cells and macrophages. APCs process the antigens and present them to T cells, initiating an adaptive immune response. This response includes the activation of B cells that produce pathogen-specific antibody responses, as well as T cells that either kill infected cells or coordinate further immune actions. The immune system also creates memory B and T cells, ensuring a rapid and potent response during future infections.

An emerging approach in vaccine development involves bacterial vector vaccines, which use live genetically modified bacteria to deliver antigens from other pathogens into the host. These bacteria, often non-pathogenic or attenuated strains such as *Salmonella*, *Listeria*, or *Lactobacillus*, are engineered to express foreign antigens. Upon entry into the body, bacterial vectors are readily recognized by PRRs, such as Toll-like receptors (TLRs), which initiate the innate immune response. These bacteria can actively invade cells, particularly APCs, delivering antigens directly into the cytosol of immune cells. This mechanism promotes a robust T cell-mediated response, in addition to B cell activation, leading to antibody production and the development of memory cells essential for long-lasting protection.

The immune system's response to pathogens is largely shaped by different types of T helper (Th) cells, with naive CD4^+^ T cells differentiating into either Th1 or Th2 cells depending on the surrounding cytokine environment [13]. Th1 cells are essential for cell-mediated immunity, which is critical for defending against intracellular pathogens like viruses and certain bacteria [14]. The presence of interleukin-12 (IL-12) and the activation of transcription factors such as T-bet drive Th1 differentiation, leading to the production of Th1-associated cytokines, particularly interferon-gamma (IFN-γ) [15]. IFN-γ enhances inflammatory responses and activates macrophages, which play a key role in clearing intracellular pathogens. Conversely, Th2 cells are involved in immunity against extracellular pathogens like helminths and play an important role in allergic reactions [16]. The Th2 differentiation is driven by IL-4, which activates the transcription factor GATA3, promoting the production of Th2-associated cytokines such as IL-5 and IL-13 [17]. These cytokines foster antibody production and recruit eosinophils, aiding in defense against extracellular pathogens.

The ability to modulate the immune response toward either Th1 or Th2 dominance by selecting specific bacterial strains has significant implications for vaccine development. Vaccines targeting intracellular pathogens may benefit from strains that induce strong Th1 responses, such as *Mycobacterium tuberculosis*, *Listeria monocytogenes*, or *Salmonella enterica*, which are suited to promoting cell-mediated immunity. Conversely, vaccines aimed at combating extracellular pathogens or managing allergic reactions may favor a Th2-skewed immune response [17]. Bacterial pathogens like *Bordetella pertussis* [18,19] and *Streptococcus pneumoniae* [20] have the potential to drive Th2-dominated immunity, characterized by robust antibody responses and eosinophilia.

Understanding the mechanisms governing Th1 and Th2 differentiation is thus critical for designing vaccines that elicit the desired immune response. By carefully selecting bacterial vectors and antigens, vaccine developers can tailor immune responses to effectively target specific pathogens. This approach holds promise not only for combating infectious diseases but also for therapeutic applications where modulating the immune response is key to treatment success.

### 1.2 Key bacterial species and their unique traits essential for vaccine development

In recent years, major strides have been made in exploiting bacterial vectors to deliver plasmids or antigens into the host cells [21]. Examples of well-studied attenuated bacteria serving as carriers for DNA vaccines are *Yersinia enterocolitica* [22], *Listeria monocytogenes* [23]*,* and *Salmonella* spp. [24], and *Shigella* spp [25]. Additionally, non-pathogenic lactic acid bacteria such as *Lactococcus* spp., *Streptococcus* spp., and *Lactobacillus* spp. have also been explored as promising candidates in DNA vaccine delivery systems. These bacterial vectors offer versatile platforms for DNA vaccine delivery, with the potential to elicit robust immune responses against targeted pathogens or antigens. Careful consideration in selecting bacterial strains is essential, as each bacterial species possesses unique immune modulatory mechanisms and intracellular lifestyles such as invasion, replication and dissemination. For example, *Salmonella* features an intravacuolar lifestyle [26] whereas *Listeria* demonstrates an intracytoplasmic lifestyle [27]. Some examples of engineered bacterial strains studied as vaccine carriers are summarized in Table 1.

**Table 1:** Examples of engineered bacterial strains as vaccine carriers.

Bacteria	Strains	Gene for Attenuation	Heterologous antigen	Test model	Remarks	Reference
*S.* Typhimurium	SL7207	*aroA*	Cruzipain (Scz)	Mice	Elicited immune response against *Trypanosoma cruzi* infection	[180]
	SV9699	Δ*aroA* Δ*aroB*	*Pseudomonas aeruginosa* antigen PcrV	Mice	Developed a vaccine expressing PcrV antigen using T3SS effector SseJ	[181]
	SL7207	*aroA*	Transmissible gastroenteritis virus N gene	Mice and piglets	Induced humoral, cellular and mucosal immune responses	[182]
	ML21/ML88	*ΔaroA, ΔpryF or ΔaroA, ΔpryF, ΔinvA*	*Staphylococcus aureus* SaEsxA and SaEsxB	Mice	Utilized the SPI-1 T3SS effector SipA; oral administration induced antigen-specific immune responses	[183]
	χ12341	ΔPmurA	*Helicobacter pylori* antigens	Mice	Employed an innovative vector strain for delivery and expression of antigens; induced humoral and cellular immune responses	[184]
	VNP20009	*purI msbB*	–	Human	Phase I study; tested for intravenous administration in patients with metastatic melanoma	[185]
	Re88	*aroA and Dam*	Ovalbumin	Mice	An H_2_O_2_ – inactivated *Salmonella* inducing IgG titer; subcutaneously vaccinated mice resulted to smaller tumors	[186]
	LH430	*phoP/phoQ*	Outer membrane proteins fusion (OprF-OprI) from *Pseudomonas aeruginosa*	Mice	Subcutaneous administration elicited higher serum IgG and IgA than intramuscular; protective response against *P. aeruginosa* infection	[187]
	χ9241/χ9277	Δ*sopB*	PspA	Mice	Induced high levels of antigen specific serum IgG and mucosal IgA	[188]
*S.* Typhi	TSB7	*aroC, ssaV*	*Escherichia coli* heat labile toxin (LT-B)	Human	A pilot human study; induced humoral immune responses	[189]
	VXM01	*galE* *thyA* Vi^−^	Vascular endothelial growth factor (VEGFR-2)	Human	Phase I trial	[190]
	ST011	*pilS, pilT*	Human immunodeficiency virus (HIV) *gag* gene; *gp120* gene	Mice	Intranasal inoculation induced antigen-specific IgG and IgA; elicited immune responses against both antigens of HIV	[191]
	Ty21a	*galE* *thyA* Vi^−^	*Helicobacter pylori* urease	Human	Showed T-cell response to *Helicobacter* urease	[192]
*Listeria* spp.	*L. ivanovii*	*dal/dat*	–	Mice	Development of balanced-lethal system	[193]
	*L. monocytogenes*	*actA/inlB*	Multi-epitope chimeric antigen (MECU) from *Helicobacter pylori*	Mice	Tested for immunotherapeutic effect on *H. pylori*-infected mice	[194]
	*L. monocytogenes*	yzuLM4Δ*act/plcB*	Fusion antigen FbpB-ESAT-6 (rLM) against *M. tuberculosis*	Mice	Induced Th1 immune response and conferred protection against tuberculosis	[195]
	*L. ivanovii*	Δ*ilo:hly*	–	Mice	Evaluated for immune protection against *L. ivanovii* in mice	[196]
*Yersinia* spp.	*Y. enterocolitica* WA_314_*sodA*- or WA *irp1*	*sodA*, *irp-1*	*Entamoeba histolytica* surface lectin	Rodents	Heterologous expression of different sections of *Entamoeba histolytica* surface lectin via T3SS for oral vaccination of rodents against amoebiasis	[197]
	*Y. enterocolitica*	*Ye sycH-*	–	Mice	Attenuated strain that induced higher *Yersinia*-specific IgA levels	[198]
	*Y. enterocolitica*	Yops	Listeriolysin (LLO) of *Listeria monocytogenes*	Mice	Tested the immunogenicity, protective efficacy and virulence of different *yop* mutants	[199]
*Vibrio cholerae*	*V. cholerae 01* strain H12	Recombinant *Vibrio cholerae* ghost	major outer membrane protein (MOMP) of *Chlamydia trachomatis*	Mice	Induced elevated local genital mucosal and systemic Th1 responses when delivered intramuscularly	[200]
Lactic acid bacteria	*Lactococcus lactis*	–	FMDV VP1 genes	Mice	Evaluated against Foot-and-mouth disease virus in mice; produced high levels of mucosal sIgA, humoral and cellular immune responses	[201]
	*Lactobacillus plantarum* (WXD234)	–	*Staphylococcus aureus* nontoxic mutated α-hemolysins	Mice	Induced mucosal immunity conferring protection against *S. aureus* pulmonary infection and reduced abscess size	[202]
	*Lactobacillus casei* strain W56	–	Bovine viral diarrhea virus (BVDV) E2 protein fused with ctxB	Mice	Induced significant levels of BVDV-neutralizing and antigen-specific antibodies	[203]
	*Lactobacillus casei pPG-COE-DCpep/L393*	–	DC-targeting peptide fused with a PEDV COE	Piglets	Elicited systemic and mucosal immune response and protects piglets against PEDV infection	[204]

Among the bacterial strains, *Salmonella* has been the most extensively investigated vaccine vector strain that stimulates robust immune responses owing to Pathogen-Associated Molecular Patterns (PAMPs) such as lipopolysaccharides (LPS) and flagellin [28,29]. Attenuated *Salmonella* vectors have increasingly gained interest in vaccine development and delivery due to their limited virulence but retained ability to stimulate the immune responses in mucosal and systemic compartments by the sustained and efficient target antigen production. One of the unique abilities exploited in the *Salmonella* system is its ability to translocate T3SS effector proteins into the host cell cytoplasm mediated by SPI-1 and SPI-2 [30]. *Salmonella* carriers typically favor a Th1 response due to their ability to reside in macrophages, leading to intracellular antigen presentation via MHC class I molecules which promote cytotoxic T cell activation. Furthermore, the activation of dendritic cells and macrophages enhances the production of IL-12, a cytokine crucial for the differentiation of naïve T cells into Th1 cells. The localization of *Salmonella* within the *Salmonella*-containing vacuole (SCV) can limit the effective presentation of expressed foreign antigens to the MHC-I pathways. However, these limitations can be addressed through genetic modifications such as deleting *sifA* gene, which facilitates the bacteria's rapid escape from the SCV, thereby enhancing antigen presentation [31]. Furthermore, the heterologous antigens are often expressed as fusion in a *Salmonella* T3SS-mediated translocation for carrier effect that can direct the delivery of the antigen to the target location in the cell for increased immunogenicity [28]. *Salmonella*-based vaccines can be engineered to induce Th2-biased responses by manipulating the type of antigen presented and the adjuvants used. Aside from its induction of balanced humoral and cellular immunity, *Salmonella* can be conveniently administered orally which induces mucosal immunity and influences Th2 induction by engaging mucosal-associated lymphoid tissues (MALT) and generating IgA antibodies. Coupled with engineered expression plasmids and biotechnological advances, *Salmonella* can also carry multiple antigens from different pathogens, allowing for multivalent vaccine development against a wide range of infectious diseases (Table 1).

On the other hand, *Listeria* has a unique ability to breach biological barriers and elicit immune responses making it an attractive candidate for vaccine delivery. This Gram-positive, intracellular bacterium is well-suited for stimulating cell-mediated immunity, particularly Th1 responses, due to its unique ability to invade host cells and survive within the cytoplasm. Since *Listeria* could invade host cells and escape from the phagosome to the cytoplasm, allowing for antigen presentation via MHC class I and MHC class II pathways. The robust stimulation of T-cell responses, particularly CD8^+^ cytotoxic T-cells through fusion proteins and foreign epitopes or proteins in *Listeria,* is crucial for its application in vaccine development against intracellular pathogens as well as cancer vaccines [32]. Their induced immune response can be modified to favor Th2 immunity by selecting extracellular antigens that drive humoral immunity or by adding Th2-promoting adjuvants. Furthermore, its capacity to traverse the mesenteric lymph node barrier and disseminate through the bloodstream underscores its potential for systemic vaccine delivery [33]. *Listeria* also employs an actin-based motility system for cytoplasmic mobility and cell-to-cell spread, facilitating its intracellular replication and subsequent immune response induction [34].

Other bacteria used for vaccine development include *Mycobacterium bovis* BCG strain and *Yersinia*. BCG has a long history of use in humans as a vaccine against tuberculosis (TB) due to its strong elicitation of Th1 immune responses, beneficial for TB and other intracellular pathogens. Besides its primary use as a vaccine against TB, BCG has been recently discovered to show some potential protection against other infectious diseases such as malaria [35,36], human immunodeficiency virus (HIV) [37] and respiratory infections including influenza [38] as well as the application as immunotherapy for bladder cancer [39], proving its promising vaccine platform against a wide range of diseases.

Attenuated *Yersinia pestis* and *Y. enterocolitica*, have also emerged as promising candidates for oral live carrier vaccines. However, unlike intracellular bacteria such as *Salmonella* and *Listeria*, *Y. enterocolitica* primarily replicates extracellularly within abdominal lymphoid tissues after crossing the intestinal barrier [22,40,41]. To facilitate the translocation of heterologous antigens into the cytosol of antigen-presenting cells (APCs), *Yersinia* can be engineered through its type 3 secretion system. Moreover, since wild-type Yersinia can cause self-limiting gastroenteritis and mesenteric lymphadenitis in humans [42], attenuation is imperative before utilizing them as oral vaccine carriers.

However, the safety concerns surrounding live-attenuated strains of pathogenic bacteria, have sparked interest in utilizing commensal and non-pathogenic food-grade bacteria like lactic acid bacteria (LAB) as vaccine vectors, addressing certain limitations associated with live-attenuated pathogenic bacterial vaccine carriers [43]. LABs, known for their suitability for oral vaccine development and their efficacy in mucosal administration, have emerged as promising candidates for vaccine delivery platforms [44,45]. Notably, strains from the *Lactobacillus* genus (e.g., *L. acidophilus*, *L. plantarum*, *L. paracasei*, *L. casei*, and *L. rhamnosus*), along with *L. lactis* [[46], [47], [48], [49]] and *Streptococcus gordonii* [50], have been genetically engineered as live bacteria vaccine vectors for delivering foreign antigens [51] (Table 1). The selection of *Lactobacillus* strains as vaccine carriers is based on their ability to colonize mucosal surfaces, express foreign antigens, and elicit immunogenic responses [52]. LABs have several advantageous characteristics as vaccine carriers. They are acid resistant, enabling them to survive the passage through the stomach and efficiently deliver heterologous antigens for immune response induction facilitating easy oral or local administration of the bacterial vaccine. Furthermore, the absence of lipopolysaccharide (LPS) in LAB eliminates the risk of endotoxic shock associated with attenuated pathogenic bacterial carriers [53]. Recent applications and studies on LABs include the nasal route inoculation of *L. lactis*, *S. gordonii*, and various *Lactobacillus* spp. which has evoked both systemic and mucosal antigen-specific immune responses in mice [54], while oral and vaginal immunizations with *L. lactis* and *S. gordonii* have also demonstrated promising outcomes [55,56].

*Streptococcus gordonii*, a gram-positive bacterium and commensal in humans colonizing oral and vaginal cavities, has emerged as a vaccine delivery vehicle due to its efficient colonization and transient persistence in the digestive tract. Notably, the Challis strain of *S. gordonii* has been extensively studied for vaccine delivery since the 1990s, with recombinant strains genetically stable *in vivo* through surface expression of resistance markers and recombinant antigens [50]. This involves chromosomal integration of recombinant DNA encoding vaccine antigens fused to the gene encoding the M6 surface protein of *Streptococcus pyogenes*, thereby anchoring the fusion protein to the bacterial surface [53]. Further advancements include co-expression of immunomodulatory molecules, such as murine IL-2 and IFN-γ, along with the target antigen, resulting in functional cytokine secretion alongside antigen delivery to the immune system [57].

*Lactococcus lactis*, utilized as a live vector for vaccines, is another promising candidate for expressing heterologous proteins and delivering antigens. Following its initial exploration as a mucosal vaccine against *Streptococcus* mutans, subsequent investigations have demonstrated its high immunogenicity, expressing various antigens such as Cu-Zn superoxide dismutase of *Brucella abortus* [46], virulence-associated protein A of *Rhodococcus equi* [58], VP4 of porcine rotavirus [59], enterotoxin B of *Staphylococcus aureus* [47], papillomavirus type 16 (HPV16) E7 protein [48], and low-calcium response V of *Yersinia pseudotuberculosis* [49]. Additionally, *L. lactis* has been evaluated as a carrier for various antigens against pneumococcal respiratory infections through different mucosal inoculation routes such as nasal, oral, broncho-alveolar lavages (BAL), and intragastric [60,61]. Various *Lactobacillus* spp. have also been extensively investigated as potential mucosal vaccine candidates, owing to their noninvasive administration, stability of genetic modifications, relatively low cost, and high safety profile [62]. Their use holds promise for oral or intranasal delivery routes, and numerous reviews have highlighted the potential of *L. lactis* as a vaccine carrier [45,48,51,62].

### 1.3 Immune modulation by pathogenic and commensal bacteria

**Fig. 1:**
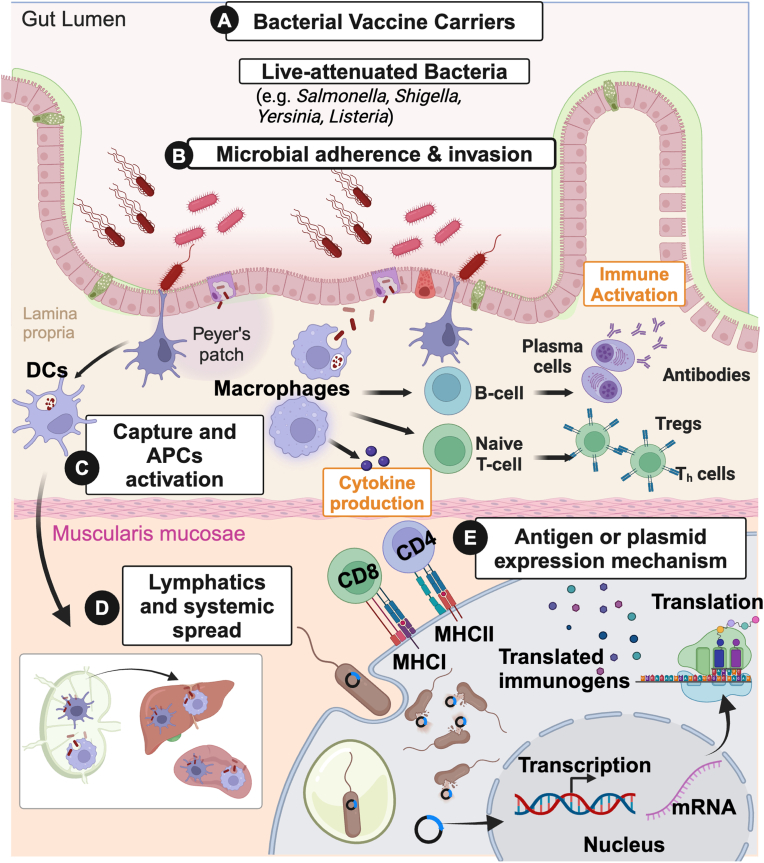
Mechanism of bacteria-based antigen delivery, expression and induction of immune response by live-attenuated bacteria (e.g. *Salmonella, Shigella, Yersinia, Listeria*), (A) inoculated orally, are directed into the gut lumen. (B) The attenuated bacterial vaccine carriers adhere and invade the intestinal epithelium and then (C) translocated from the gut lumen through M cells or captured by macrophages or dendritic cells. (D) The antigen-presenting cells (APCs), such as macrophages and dendritic cells, are then activated leading to cytokine production and immune activation. Furthermore, (D) APCs invaded with bacterial vectors can spread to different organs such as the liver and spleen through the lymphatics and bloodstream (E) When bacterial vectors (e.g. *Salmonella)*. invade the host cells, the plasmid DNA carrying the gene encoding the immunogen is released into the cytoplasm through bacterial lysis. The delivered plasmid undergoes transcription in the cell nucleus followed by translation of the mRNA outside the nucleus into immunogenic proteins which are then presented to CD8^+^ and CD4^+^ cells via MHCI and MHCII, respectively. This will then elicit T-cell responses. In addition, DCs can also present antigens to B cells to differentiate into specific antibodies (Created in Biorender.com).

The immune reactions generated by pathogenic bacteria and commensal bacteria differ in several important ways. Pathogenic bacteria often trigger a strong and immediate immune response, characterized by significant induction of inflammatory responses. This activation is modulated by various cytokines, and chemokines, that lead to the migration of immune cells, DCs, neutrophils, macrophages, etc. leading to the activation of innate and adaptive immune responses. The activation of T and B cells leads to the secretion of antigen-specific cell-mediated and humoral responses for rapid clearance of the invading pathogens while creating a long-lasting memory response [63,64] (Fig. 1). Pathogen-associated molecular patterns (PAMPs) act as critical signals for the host's immune system, detected by a sophisticated array of pattern recognition receptors (PRRs). While bacterial species used in vaccine delivery strategies are not entirely stripped of their virulence, they are engineered to retain attenuated virulence, ensuring safety but still possess key structural components such as lipopolysaccharides (LPS), lipoteichoic acid, flagellin, and pili that maintain their potent immunostimulatory properties. Since these bacterial components are recognized by the immune system, they have been of great interest for vaccine adjuvants.

For instance, the LPS is a powerful activator of Toll-like receptor 4 (TLR4) [65], initiating downstream signaling cascades that lead to inflammation and immune activation. While LPS could be too toxic for use as an adjuvant, a detoxified derivative of LPS, the monophosphoryl lipid A (MPLA) still retains its immunostimulatory properties [66] which are applied in human papillomavirus (HPV) vaccine [67]. On the other hand, flagellin specifically engages Toll-like receptor 5 (TLR5) which plays a key role in innate immunity, stimulating robust mucosal and systemic immune responses [68]. A recombinant flagellin-based influenza vaccine has been tested in clinical trials [69]. Moreover, the bacterial DNA contains unmethylated CpG motifs that are recognized by TLR9, leading to the activation of immune cells such as B cells and dendritic cells [70]. These motifs have been synthetically mimicked in CpG oligodeoxynucleotides (CpG-ODN) and are incorporated into vaccines such as Hepatitis B vaccine [71]. Other key bacterial components being evaluated as adjuvants include the components of bacterial cell walls, such as the peptidoglycan and its breakdown product muramyl dipeptide (MDP) which are potent stimulators of the innate immune system. These MDP derivatives have been tested in vaccine formulations as immune stimulants and are still under evaluation [72]. Certain bacterial polysaccharides like the Zwitterionic polysaccharides found in *Streptococcus pneumoniae* and *Bacteroides fragilis*, are under investigation as an adjuvant specifically to enhance T-cell-mediated immune responses [73,74].

These bacterial components can be fractioned using biochemical techniques such as detergent solubilization and chromatography that are then further purified [75]. The genes encoding the bacterial components can also be cloned into recombinant expression systems to produce isolated immune modulatory molecules like flagellin and MPLA in more controlled conditions and allow modification of these components to reduce toxicity while retaining their immune-stimulating properties. Synthetic oligonucleotides such as CpG-ODN are also designed to mimic the immune-modulatory properties of bacterial DNA. Similarly, synthetic analogs of MDP are created to improve stability and reduce side effects in vaccine formulations [76].

Upon systemic administration, bacterial species like *Salmonella* and *E. coli* proficiently infiltrate the mucosal surfaces of the host, with a certain portion entering the systemic circulation via lymphoid tissues or the blood circulatory system. Once they are detected by the host's professional antigen-presenting cells such as dendritic cells (DCs) and macrophages, intricate immune activation commences, eliciting a highly specific adaptive response involving CD4^+^ and CD8^+^ T cells alongside antibody-producing B-cell lineages [77]. This immune regulation within the host is finely regulated by molecular messengers such as cytokines and chemokines, orchestrating signaling pathways of feedback loops to prevent the onset of uncontrolled pro-inflammatory responses and mitigate potential tissue damage.

Moreover, bacterial vector-mediated immune responses boost the natural predilection for targeting specific cell types within the host's immune system. For instance, *Salmonella* exhibits a preference for proliferating within macrophages, prime candidates for antigen presentation. Consequently, engineered immune responses can be directly channeled to antigen-presenting cells, expediting deliberate activation against target antigens. Additionally, diverse bacterial species harbor their unique intracellular or extracellular niches for proliferation, offering unparalleled maneuverability in tailoring immune responses to precise locations within the host's internal environment. Moreover, many bacterial strains employed in vaccine studies are intestinal pathogens or commensals, ideally primed for eliciting mucosal immune responses, thus facilitating convenient oral administration. Post-traversing the intestinal barrier, the bacterial vector promptly encounters antigen-presenting cells for processing and presentation within lymphoid tissues, culminating in the activation of T cells and subsequent B-cell proliferation, crucial for pathogen neutralization and the development of immunological memory.

**Fig. 2:**
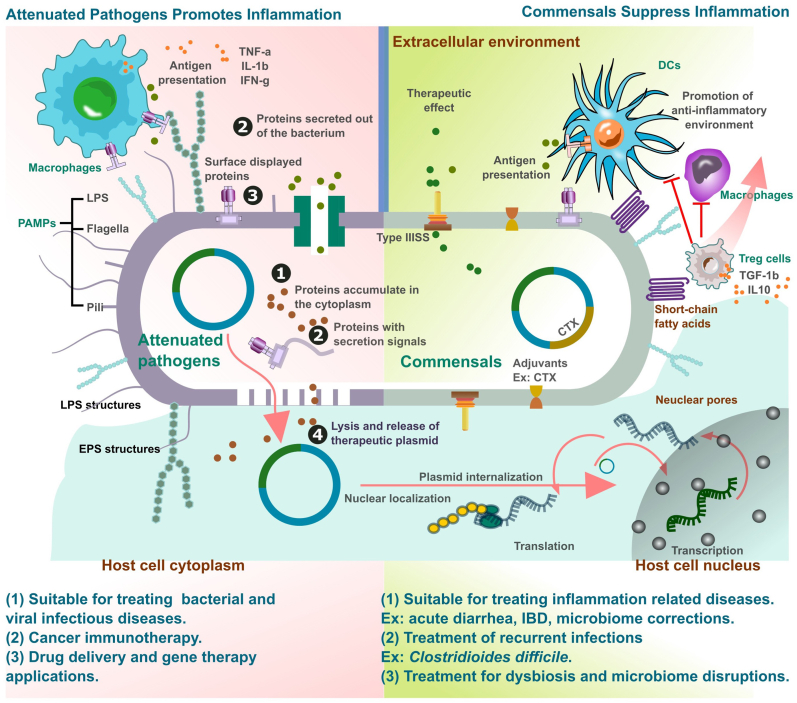
Immune modulation mechanisms of attenuated pathogens and commensal bacteria and their potential prophylactic and therapeutic applications. Attenuated pathogens, capable of displaying or secreting antigen loads, trigger robust immune responses by inducing antigen-presenting cells (APCs), like macrophages, to secrete cytokines and chemokines. On the other hand, commensal bacteria which can also be genetically engineered to present heterologous antigens offers therapeutic benefits particularly in managing inflammatory conditions by promoting anti-inflammatory environment, largely through the mediation of anti-inflammatory cytokines released by regulatory T cells (Tregs).

Conversely, commensal bacteria, which are naturally present in the mucosal surface and beneficial to the host, induce a more subdued immune response. The immune system tolerates the commensal bacteria as they maintain homeostasis and provide protection against pathogenic bacteria by competing for resources. These bacteria species deploy T-regulatory cells (Tregs) and promote anti-inflammatory cytokines (Fig. 2), thus maintaining immune homeostasis and halting the overactivation of immune responses that could lead to autoimmune diseases [78]. The interaction between commensals and the host immune system can also shape the adaptive immune system, enhancing the host's ability to respond to pathogens while minimizing unnecessary activation of inflammation. Certain commensal bacteria possess intrinsic immune-modulatory properties and thus, can be used to enhance the immune response to vaccines by acting as adjuvants. For example, the *Lactobacillus rhamnosus* GG strain has been used as an adjuvant that enhances the production of IgA antibodies [79]. Moreover, the zwitterionic polysaccharides (ZPS) produced by *Bacteroides fragilis* have been shown to promote the development of regulatory T cells (Tregs) [80].

Therefore, the selection of bacteria species for personalized vaccine development, whether attenuated pathogen or commensal bacteria can be decided based on the specific prophylactic or therapeutic requirements. For example, owing to the robust induction of immune responses by attenuated bacteria, they could be suitable for vaccine development against infectious diseases, cancer immunotherapy, and gene therapeutic approaches. On the other hand, commensal bacteria can be utilized in delivering heterologous antigens and help restore gut health which is critical for treating various inflammatory conditions, such as acute diarrhea, inflammatory bowel disease (IBD), and chronic conditions [81]. Clinical studies have demonstrated that the commensal bacteria species can be successfully utilized in bacteriotherapy against recurrent infections such as *Clostridioides difficile*, using techniques such as fecal microbiota transplantation [81].

## 2 Advanced bacterial engineering approaches for safe vaccine applications

**Fig. 3:**
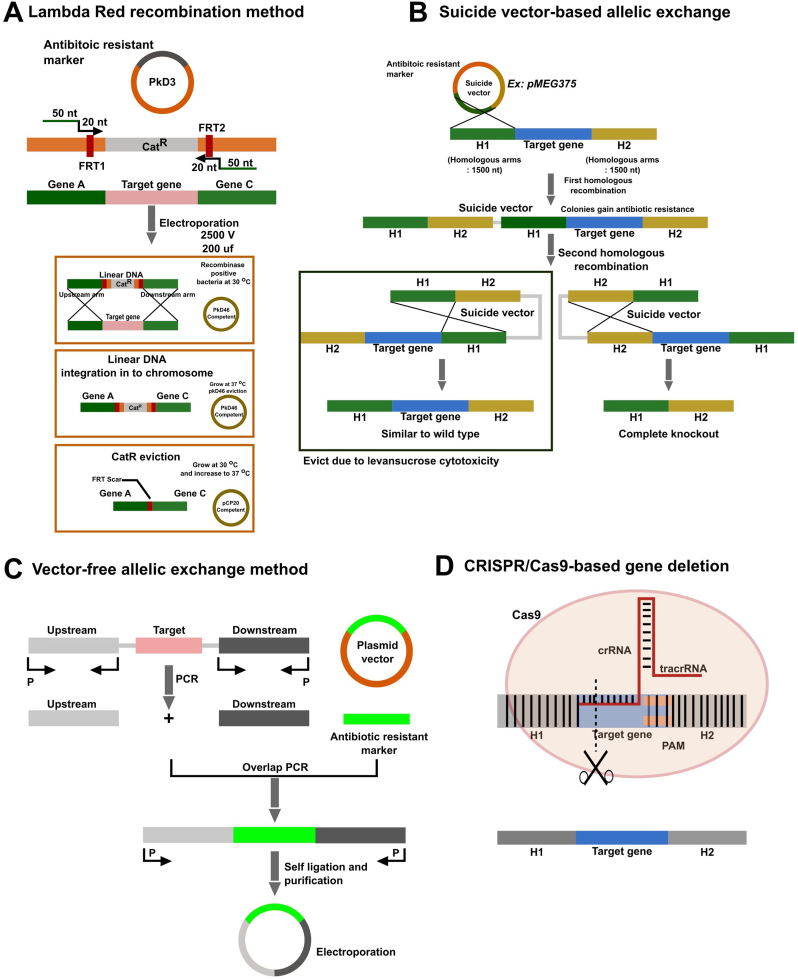
Genetic methods employed in gene deletion and mutagenesis. (A) Lambda Red recombination method. Long flanking primers (70 nt) is used to amplify CatR resistant gene (CatR for pKD3 and KanR for pKD4) and the PCR product is transformed into pkD46 transformed recombinant competent host bacteria. Successful integration of linear segment demarcates single-step genetic replacement of target gene by CatR gene. Subsequently, CatR gene can be evicted using the help of pCP20 plasmid which encodes flipase enzyme that target FRT sequences encoded into linear DNA segment. Both pKD46 and pCP20 are temperature sensitive and must be grown at 30 °C. (B) Allelic exchange employs a suicide vector (Ex: pMEG375) to harbor long (1500 nt) flanking regions. Allelic exchange utilizes a two-step recombination process, whereby the first recombination will incorporate plasmid components into the host chromosome. Complete eviction of the plasmid backbone and target gene occur along the second recombination step. To identify double recombinants, sucrose is used in the screening process, as sucrose does not support the growth of single recombinants due to levan sucrose toxicity that is produced by the plasmid-encoded levansucrase enzyme. (C) Similar to allelic exchange method, long upstream and downstream flanking regions of the target gene are amplified and are allowed to self-ligate into a circular plasmid construct. Screening is based on antibiotic resistance. (D) Novel CRISPR/Cas9 systems can be adapted to delete target genes from bacteria chromosomes based on gRNA sequence. Target specificity is governed by gRNA (crRNA) sequence and the presence of PAM sequence. Abortion of gene function occurs due to frame-shift mutations.

Recent molecular advancements have revolutionized the genetic and chemical manipulation of biological systems in bacteria, enabling the safer and more effective presentation of target antigens to host tissues [[82], [83], [84]]. These strategies include various techniques such as genetic manipulations, chemical mutagenesis, and recombinant DNA technology to attenuate pathogenic bacteria, often involving targeted gene deletions in virulence regulatory systems or metabolic pathways [5,83,85]. The delicate balance between bacterial pathogenicity and immunogenicity is crucial for ensuring bacterial survival and replication within the host [86]. In the subsequent sections, we review the different bacterial attenuation methods employed in recent studies (Fig. 3).

### 2.1 Deletion of virulence genes

Genetic methods play a pivotal role in the development of live-attenuated bacterial vaccines, particularly through the deletion or modification of virulence genes critical for pathogenesis or metabolic pathways. It is crucial to have a comprehensive understanding of the virulence factors essential for bacterial pathogenicity. With the rapid expansion of genomic and proteomic data, researchers now have immense opportunities to identify these virulence factors across various bacterial species [87]. Once the functions of these virulence genes are determined, genetic engineering techniques are employed to delete or knock out these genes from the bacterial genome [88,89]. Various curated techniques for gene deletion in bacterial genomes are available such as Lambda red recombination [90] (Fig. 3A), allelic exchange method (Fig. 3B) [91], vector-free allelic exchange methods (Fig. 3C) [92], and the latest CRISPR/Cas9 mediated strategies (Fig. 3D) [93]. These methods have inherent advantages and disadvantages, depending on factors such as the target host, genes of interest and environmental conditions.

Lambda red recombination involves the use of homologous recombination machinery encoded by bacteriophage lambda to facilitate gene replacement or deletion [90] (Fig. 3A). This technique allows for the rapid and efficient introduction of desired mutations into bacterial genomes, including the deletion of virulence genes in pathogens like *Salmonella*. On the other hand, suicide vector-mediated allelic exchange relies on the delivery of a recombinant suicide vector containing regions homologous to the target gene into the bacterial cell, followed by recombination events resulting in gene deletion [94] (Fig. 3B). Similarly, vector-free allelic exchange methods utilize linear DNA fragments containing homologous sequences to replace target genes through recombination [95] (Fig. 3C). Recently, CRISPR-Cas9-mediated techniques have emerged as powerful tools for precise genome editing, enabling targeted gene deletions in bacterial genomes with high efficiency and specificity [96] (Fig. 3D). These gene deletion techniques contribute significantly to bacterial vaccine development by enabling the generation of attenuated strains with enhanced safety profiles and immunogenicity.

### 2.2 Chemical methods (chemical mutagenesis)

Chemical methods offer alternative approaches to genetic manipulation for attenuating pathogenic bacteria, rendering them safer for vaccine development and therapeutic applications. These methods involve the use of various compounds or chemicals to reduce bacterial virulence, ensuring safety without extensive genetic modification. Nitrosoguanidine or ethyl methanesulfonate are commonly used chemical mutagens that induce random mutations throughout the bacterial genome, disrupting virulence genes or essential metabolic pathways [97]. With the introduction of these mutations, bacteria become less pathogenic, offering a rapid means of generating attenuated strains. Additionally, chemical inhibitors such as rifampicin or streptomycin can selectively target specific virulence factors or essential pathways vital for bacterial survival and pathogenesis [98,99]. By inhibiting bacterial protein synthesis or RNA polymerase activity, these chemical inhibitors disrupt essential cellular processes while preserving the bacteria's immunogenicity. These chemical methods of bacterial attenuation offer advantages in ease of implementation and scalability. However, they may result in numerous unrecognized or unintended mutations throughout the genome. Notable examples of bacterial attenuation via chemical methods include the generation of the *Salmonella* Typhi Ty21a strain (GalE-negative) and various *M. bovis* strains [[100], [101], [102]].

### 2.3 Attenuation by radiation

Irradiation of bacteria represents a promising method of attenuation in vaccine development. This approach prevents bacterial replication while retaining metabolic activity, leading to higher humoral immune responses and protection against bacterial infections in both humans and animals [103]. Irradiated bacterial vaccines have been successfully developed from a wide range of Gram-negative and Gram-positive bacteria, including *Mycobacteria*, *Bacillus* Calmette-Guerin, *Salmonella*, *Brucella*, *Vibrio*, *Streptococcus*, and *Listeria* [103]. Lethal *γ*-irradiation has been observed to preserve the immunomodulatory properties of probiotic bacteria in a murine model of inflammatory bowel disease, suggesting that irradiated bacteria retain adjuvant and antigenic structures that induce immunogenicity [104,105]. For instance, genetically attenuated *Listeria monocytogenes* mutants have been successfully generated through inactivation with ultraviolet light while retaining metabolic activity necessary for immunogenicity [106]. Studies have shown that irradiated *Listeria* induces protective T-cell immunity [105]. Additionally, vaccination with a genetically attenuated *Staphylococcus aureus* mutant inactivated by UV irradiation has been shown to increase survival and decrease bacterial burden in the mice challenged with virulent methicillin-sensitive and MRSA-challenged mice, indicating induction of humoral immunity [107].

Another alternative method for attenuating bacteria in vaccine development involves the use of ionizing radiation, such as electron beam (eBeam) technology. This cutting-edge approach harnesses near-speed-of-light electrons generated from electricity as a non-radioactive source of ionizing radiation [108]. Unlike traditional methods involving radioactive sources, eBeam technology offers a safer and more controlled means of bacterial attenuation. Exposure of microbial cells to ionizing radiation induces direct or indirect effects that result in their inactivation. The collision of electrons within microbial cells leads to the breakdown of nucleic acids and the production of radiolytic species, causing damage to the cells and potentially introducing lethal mutations in their nucleic acids [108,109]. Studies involving eBeam irradiation of bacteria such as *S*. Typhimurium and *S*. Enteritidis have demonstrated promising results in evaluating the effectiveness of immunomodulators in chicken models [110]. These studies have shown that eBeam-irradiated bacteria can protect against virulent challenges of *Salmonella*, both in intramuscularly vaccinated laying hens and in chickens vaccinated *in ovo* with eBeam-irradiated *S*. Typhimurium [111].

## 3 Advanced engineering strategies for vaccine antigen delivery and antigen presentation by bacteria

### 3.1 Chromosomal integration and plasmid promoter systems

**Fig. 4:**
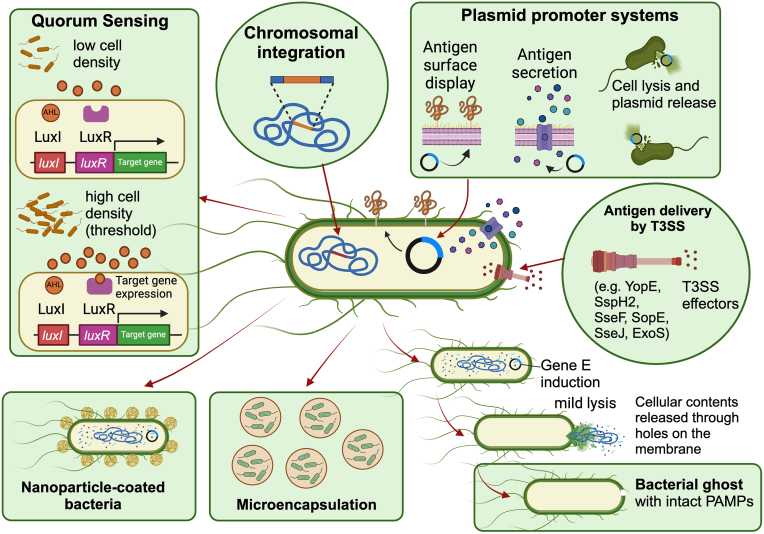
Advanced strategies for vaccine antigen delivery and presentation by bacteria. The figure illustrates the various strategies employed for exploiting bacteria for vaccine development and antigen delivery. Such strategies include chromosomal integration, plasmid promoter systems, T3SS-mediated antigen delivery, bacterial ghosts, quorum sensing, nanoparticle-coated bacteria and microencapsulation.

**Fig. 5:**
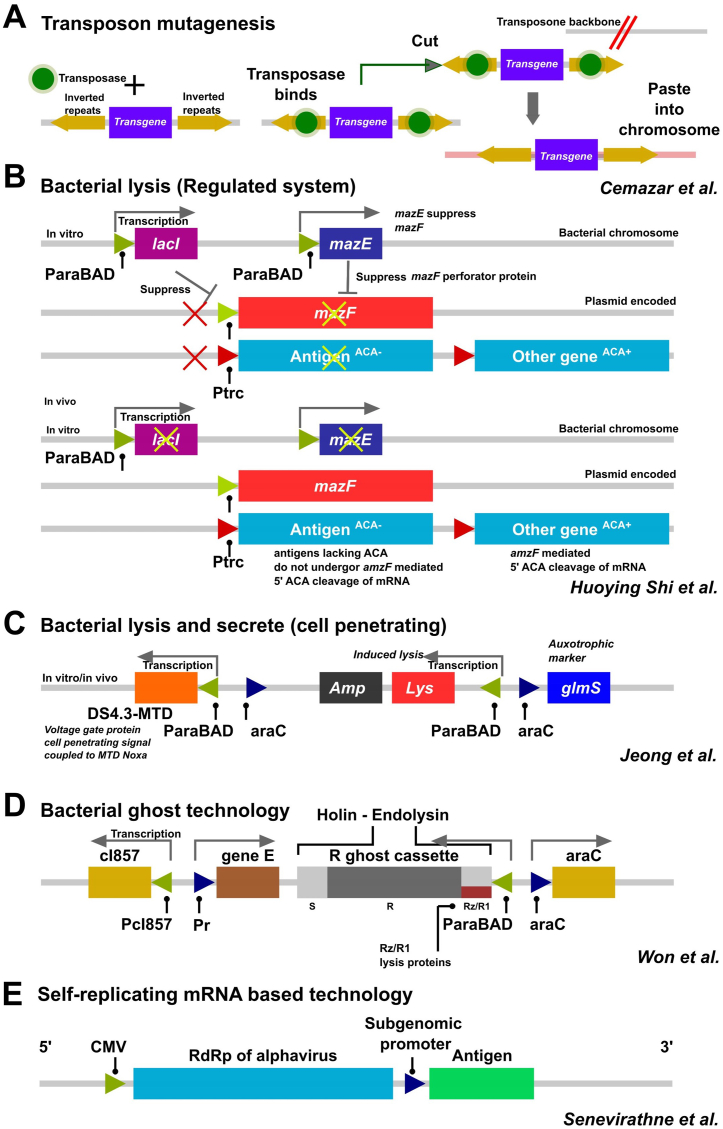
Antigen expression technologies. (A) Transposon mutagenesis. Random integration of antigens into bacterial chromosomes can be achieved using transposon technology. Typical transposon cassettes include a target gene flanked by two inverted repeats. The essential enzyme transposase is provided by a supporting plasmid. The site of integration is obtained by rescue cloning and sequencing procedures. (B) Regulated lysis systems. The diagram depicts the antigen-expressing lysis system based on *mazF* interferase gene. It is known to cause cellular perforations, aiding the release of bacterial content. Interferase activity consists of 5′ degradation of ACA sequence from mRNA, hence any gene coding for the particular motif degrades and sequences absent of these codons will be produced in high quantities. Unwanted expression of *mazF* gene is achieved by the arabinose-regulated *mazE* suppressor gene. (C) Bacteria encoding cell penetrating peptides. An arabinose-regulated system of lysis gene expression is demonstrated. Two selective markers have been used, *ampR* and *glmS* (auxotrophic marker) to prevent plasmid loss in *glmS* deleted mutant strains. The particular plasmid construct has been developed as an anti-cancer therapy for tumor-specific delivery of mitochondrial domain (MTD) of the Noxa gene along with a DS4.3 signal peptide derived from voltage gate protein. (D) Bacterial ghost systems. Elements for temperature-regulated ghost generation systems are depicted. cI857 temperature regulation element prevents expression of phage lysis gene *E*. A leaky expression of gene *E* was efficiently prevented by antisense RNA technology induced by an arabinose induction system using the araC-ParaBAD system. Gene *E* product passage into the bacterial peptidoglycan layer was achieved by holing and endolysin modular elements. (E) Self-replicating mRNA technology. To improve mRNA copy number upon plasmid delivery, an RNA dependent RNA polymerase (RdRp) of alpha virus can be adapted. RdRP along with subgenomic promoter is cloned into a vector. Target antigen ORFs are placed downstream of the sub genomic promoter. A massive improvement in mRNA levels has been experimentally demonstrated.

When bacteria are engineered to deliver antigens, the antigen-encoding open reading frames (ORFs) can be inserted into either bacterial chromosomes or appropriate plasmid vectors (Fig. 4). When antigen ORFs are integrated into the host chromosomes, they achieve higher genetic stability, but this occurs at a lower copy number. Hence, the level of gene expression must be manipulated by selecting a suitably strong promoter for the intended purpose. Chromosomal integration is achieved by replacing or deleting genetic loci with a genetic element containing the heterologous gene [112]. This necessitates the selection of appropriate loci without damaging essential genetic elements. On the other hand, techniques such as transposon-mediated genetic integrations could cause problems by jumping from one locus to another or random integration into multiple locations [113] despite their wide applications in mutational investigations of bacterial genomes (Fig. 5A) [114]. Alternative to chromosomal integration, the plasmid-based expression offers a versatile option for carrying heterologous antigens, with a plethora of available expression systems tailored for diverse applications (Fig. 4). However, two significant challenges must be addressed to achieve the necessary balance between immunogenicity and genetic stability. Firstly, the metabolic burden associated with plasmid replication can lead to over-attenuation of the vaccine carrier, potentially diminishing immunogenicity [86]. Additionally, the frequent spontaneous loss of plasmids can result in plasmid-less bacteria rapidly outcompeting plasmid-bearing counterparts, becoming the dominant population in tissues [115,116]. To enhance plasmid retention, various mechanisms have been proposed, including the use of self-transferring plasmids for mobilizing plasmids between bacteria, providing selective advantages to promote plasmid inheritance, implementing self-regulating origins for plasmid replication, facilitating plasmid distribution via active partitioning mechanisms, and implementing post-propagation strategies to eliminate plasmid-less bacteria [112]. However, it is crucial to note that regulatory authorities strongly discourage the use of self-transferring plasmids and clinically relevant antibiotics as selective pressures due to potential safety concerns.

A highly effective method for preventing gradual plasmid loss is to establish a conditional lethal host-vector system using auxotrophic markers. In this case, a gene that is essential for bacterial survival is deleted from its genome and supplemented into the plasmid in use. This innovative method does not require medically controversial selective markers such as antibiotic genes. Thus, this system does not confer a risk of antibiotic resistance. A commonly used auxotrophic marker is aspartate semialdehyde dehydrogenase (*asd*), which is an enzyme essential for amino acid synthesis and cell wall biogenesis. Without a functional *asd* gene, bacteria cannot survive and must therefore receive this gene through a plasmid designed to be stably maintained within the bacterium [86]. Typically, bacteria that lack the *asd* gene are grown in culture media supplemented with diaminopimelic acid (DAP) to supply the essential components for their survival. Successful implementation of auxotrophic lethal host-vector systems has been reported in many studies such as *S.* Typhimurium that deliver heterologous antigens such as *Lawsonia intracellularis* antigens [117], L7/L12 and BLS fusion antigens of *Brucella* [118], and the SARS-CoV-2 S1 subunit [119].

### 3.2 Antigen delivery via T3SS

Some bacteria, such as *Salmonella*, utilize T3SS to inject their proteins into host cells, enabling survival and replication within these cells [120]. Type III secretion systems (T3SS) are specialized molecular machinery used by pathogenic bacteria to inject proteins, called effectors, directly into host cells. These effectors manipulate host cell processes to promote bacterial survival, immune evasion, or intracellular replication. Scientists have leveraged this mechanism to deliver antigens and vaccines, stimulating robust immune responses. Pathogenic bacteria possess secretion systems that can deliver specific effector proteins into the cytosols of eukaryotic cells, with the T3SS being a highly conserved nanomachinery among Gram-negative bacteria. Activation of T3SS and effector expression occurs upon contact with host cells or in low calcium environments [121]. The T3SS injectisome, resembling a syringe-like nanomachine, punctures the host cell membrane to deliver and translocate effectors into the host cytosol. This makes T3SS an attractive tool for delivering antigens or proteins into target cells, making it highly sought after for medical applications like vaccine development. The delivery of heterologous protein substrates into human cells via T3SS was initially demonstrated [122] using *Y. pseudotuberculosis*, where the adenylate cyclase was fused with the N-terminal amino acids of the naturally injected effector YopE. Furthermore, the T3SS bacterial secretion system was evaluated for vaccination purposes by inserting short viral nucleoprotein epitopes of the lymphocytic choriomeningitis virus into the *Salmonella* effector SptP, resulting in protection against lethal doses of the virus in orally immunized mice [123]. This approach has also been applied for vaccination or immunotherapy using T3SS components such as SspH2, SseF, SopE, SseJ, or ExoS from other bacterial carriers like *S.* Typhimurium, *Y. enterocolitica*, and *P. aeruginosa* against various pathogens or targeting tumors [124].

### 3.3 Regulated delayed attenuation

Bacterial vaccine strains can be strategically engineered with regulated delayed attenuation and programmed self-destructing features to optimize their ability to colonize host tissues and facilitate the release of bacterial cell contents, including heterologous antigens upon lysis. Notably, recombinant attenuated *Salmonella* vaccines (RSAVs) have been developed to exhibit regulated delayed expression of attenuation mechanisms, recombinant antigens, and controlled lysis [125]. In a related seminal study, vaccine strains were engineered to exhibit a wild-type phenotype during immunization but undergo attenuation post-colonization of host tissues [126]. This was achieved through the deletion of the *pmi* gene, encoding the 6-phosphomannose isomerase essential for O-antigen synthesis [127]. The Δ*pmi* mutants can synthesize complete O-antigen when supplied with mannose, promoting efficient colonization of lymphoid tissues. However, in the absence of non-phosphorylated mannose in host tissues, O-antigen synthesis is halted, leading to delayed attenuation [126].

Another innovative approach to regulated delayed attenuation involves replacing the promoters for *fur* or *crp* with an *araC PBAD* cassette, a system regulated by arabinose [128]. This enables the controlled expression of these genes, allowing the strain to maintain a wild-type phenotype in the presence of arabinose while achieving full attenuation upon depletion of arabinose post-colonization. Furthermore, regulated delayed *in vivo* synthesis of heterologous immunogens was accomplished by integrating a chromosomal lactose repressor gene (*lacI*) under the control of the *araC pBAD* promoter [129]. In the absence of arabinose, repression of the *araC pBAD* promoter reduces the LacI concentration, thereby enhancing the synthesis of heterologous antigens under the *trc* promoter. The same *araC pBAD* regulon has been employed to increase target antigen concentration and efficient release by modulating interferase (*mazF*) from *E. coli*. Antigens lacking the 5′-ACA motif survive while antigens with the 5′-ACA motif undergo mRNA degradation. Expression levels of *mazF* are regulated by the ParaBAD-regulated *maze* gene. While this strategy is focused on perforating the bacterial membrane to release bacterial intracellular contents, some strategies utilize complete bacterial lysis systems (Fig. 5B). Laniewski et al. (2013) engineered an *S*. Typhimurium strain χ9718 with regulated delayed attenuation *in vivo*, producing *Campylobacter jejuni* CjaA protein, and assessed its immunogenicity and protective efficacy in chickens via oral administration. While robust immune responses against CjA were elicited, the reduction in *Campylobacter* colonization was not statistically significant, suggesting the potential for further enhancement through the design of a multicomponent vaccine strain [130].

### 3.4 Quorum sensing

Quorum sensing (QS) stands as a pivotal mechanism governing various bacteria processes such as bioluminescence, sporulation, antibiotic production, biofilm formation, and virulence factor secretion. This intricate communication system relies on signaling molecules known as autoinducers (AI) to coordinate gene expression within bacterial populations [131]. As the bacterial density increases, AIs accumulate, triggering alterations in gene expression that collectively regulate cellular processes. Exploiting this mechanism, bacteria can be engineered to synchronize lysis at a quorum threshold, facilitating the release of genetically encoded cargo-targeted immunotherapy [132].

The pioneering work on QS originated from *V. fischeri*, a bioluminescent marine symbiont, showcasing the regulatory dynamics of LuxI and LuxR proteins in controlling bioluminescence [133,134]. LuxI synthesizes N-(3-oxohexanoyl)-homoserine lactone (AHL), while LuxR acts as the receptor and transcriptional activator for the luxCDABE operon. At low bacterial density, LuxI is expressed basally but upon reaching a critical threshold, LuxR binds AHL, activating *luxICDABE* promoters [135] (Fig. 4). This feedback loop potentially fosters synchrony as cells transition to a high-density QS mode. This potential of quorum sensing for antigen amplification has been harnessed by researchers for developing bacterial vector vaccines. For instance, researchers developed an *in vivo* expression circuit called ironQS that responds to changes in iron availability [136]. By integrating the Fur box with luxI and luxR from *V. fischeri* and coupling it with iron uptake regulons, ironQS enables cell density-dependent expression of heterologous proteins *in vivo*. The Fur-iron complex represses the quorum sensing circuit during *in vitro* cultivation, but liberation from iron-mediated repression *in vivo* activates antigen production in the host. Demonstrating versatility, ironQS was successfully implemented in various bacterial hosts, including *E. coli*, *V. anguillarum*, *S.* Typhimurium, and *Staphylococcus aureus* [136].

### 3.5 Bacterial ghosts

The Bacterial Ghost (BG) system represents a revolutionary approach to vaccine delivery, capitalizing on the intrinsic adjuvant properties of bacteria and their versatile carrier functions for heterologous antigens [137]. This innovative system is achieved through protein E-mediated lysis in Gram-negative bacteria, facilitated by the expression of cloned gene *E* from bacteriophage PhiX174. The resulting empty bacterial envelopes retain morphological and structural features, serving as ideal platforms for vaccine delivery, particularly as carrier transport systems that enhance immunogenicity and antigen delivery efficiency [138] (Fig. 4).

What sets BG systems apart is their ability to load the inner space with peptides, drugs, DNA, or a combination thereof, enabling efficient internalization by APCs such as macrophages and dendritic cells (DCs), as well as other cell types including Caco-2 cells and HCDECs. Upon internalization, the contents of BGs are released into the cytoplasm of target cells, facilitating cross-presentation of the antigen to APCs and subsequent activation of immune responses [139]. Moreover, BGs offer the flexibility to tether foreign target antigens to their outer membrane (OM) or inner membrane (IM) or express them as S-layer fusion proteins. In a seminal study by Jechlinger (2005) [140], *E. coli* ghosts carrying the Hepatitis B virus core 149 protein anchored in either the inner or outer membrane induced significant immune responses against the foreign target antigen in mice, underscoring the versatility and efficacy of the BG system. Furthermore, the BG system has demonstrated effectiveness as a delivery platform for DNA vaccines, offering a promising avenue for the development of novel vaccine formulations with enhanced potency and safety profiles. With their unique combination of adjuvant properties and carrier functions, BGs represent a paradigm shift in vaccine delivery, offering unprecedented opportunities for the development of next-generation vaccines against a wide range of infectious diseases and cancers. Since the production of bacterial ghosts mainly involves biological methods such as genetic engineering, it avoids harsh conditions, allowing for the preservation of most bacterial epitopes. This application of genetic engineering in bacteria enables the simultaneous generation of bacterial ghost strains and surface display of antigens, facilitated by temperature-regulated elements.

These temperature-regulated genetic elements have been previously utilized to construct an innovative plasmid system for the *Salmonella* bacterial ghost strain [141]. In this system, the lysis gene *E* is regulated by *λPr*, and its expression is suppressed by a *cI857* suppressor at temperatures lower than 30^o^C (Fig. 5D). To avoid the leaky expression of lysis gene *E*, which might cause abrupt lysis, it can effectively be prevented by *araC paraBAD-*mediated expression of antisense mRNA of gene *E*. Hence, the growth phase of the bacterium can be carried out at low temperatures (30^o^C) in the presence of L-arabinose in the medium. Once the desired bacterial population is reached, replacing the medium with one that lacks L-arabinose and increasing the growth temperature to 42^o^C activates gene *E*, which generates a bacterial ghost strain [142].

### 3.6 Self-replicating mRNA-mediated antigen delivery

Recent advancements in self-replicating mRNA (srRNA) technology have revolutionized the field of vaccinology, offering promising avenues for vaccine development against various infectious diseases, including COVID-19. Self-replicating mRNA vaccines are engineered to replicate and produce antigen proteins within the host cells, triggering robust immune responses. These vaccines offer several advantages over conventional mRNA vaccines, including enhanced antigen expression, prolonged protein production, and potentially lower vaccine doses. One notable development in srRNA technology is the design of lipid nanoparticle (LNP)-encapsulated srRNA vaccines, which facilitate efficient delivery of srRNA into target cells and protect the srRNA from degradation by extracellular ribonucleases [143]. This delivery system has been instrumental in the successful development of srRNA vaccines against COVID-19, such as the Pfizer-BioNTech and Moderna vaccines, which have demonstrated high efficacy in clinical trials [144].

Furthermore, recent research has focused on optimizing the design and delivery of srRNA vaccines to enhance their immunogenicity and stability. For example, modifications to the srRNA sequence, such as the incorporation of modified nucleosides or codon optimization, can improve translation efficiency and reduce innate immune activation [145,146]. Additionally, the development of novel delivery platforms, such as synthetic mRNA replicons or viral vectors encoding srRNA, holds promise for expanding the applicability of srRNA vaccines to a broader range of pathogens [147] (Fig. 5E). Notedly, srRNA technology has also been recently incorporated into bacterial systems, such as the RdRp vector system developed by Senevirathne et al., in 2021 (Fig. 5E) [148].

### 3.7 Genetic engineering of commensal bacteria

Unlike pathogenic bacteria that require attenuation for the safe delivery of vaccines, the manipulation of commensal bacteria for vaccine purposes involves approaches that exploit their non-pathogenic characteristic and natural occurrence in the host's microbiota to either deliver pathogens or modulate the immune systems to enhance vaccine efficacy. There are various strategies for the genetic engineering of commensal bacteria such as engineering them to express heterologous antigens from pathogenic bacteria, display the antigen on their surface, secrete vaccine antigens directly into APCs by utilizing bacterial secretion systems such as Type III secretion systems (T3SS), express or overexpress natural adjuvants of bacteria such as PAMPs and through genetic modifications of the commensals that enhances their colonization at mucosal sites and stability within the host.

Since commensal bacteria colonize the host without causing disease, recombinant commensal bacteria can be engineered to express antigens from pathogens. For example, *Lactobacillus casei* has been engineered to express antigens from Rotavirus which induced protective immunity when administered orally [149]. *Lactobacillus* strains are engineered to express tetanus toxin fragment C (TTFC) which when administered via mucosal route, will induce antigen-specific IgG and IgA antibodies against tetanus [150]. *Bifidobacterium longum* has been used to deliver *Clostridium difficile* toxin B fragment against *C. difficile* infection [151].

In contrast to pathogenic bacteria which naturally possess secretion systems that can deliver the proteins into host cells, commensal bacteria can be engineered with T3SS such as those found in *Yersinia, Salmonella* and *Shigella* to deliver the antigens into the cytoplasm of APCs, facilitating MHC class I presentation and induction of T cells responses. For instance, probiotic *E. coli* strains were outfitted with T3SS to modify their antigen delivery [152]. Moreover, by engineering commensal bacteria to express or secrete adjuvants alongside the antigen, the overall immunogenicity of the vaccine can be enhanced [153]. Surface components of commensal gut bacteria such as capsular zwitterionic polysaccharides (CSPz) are also regarded as important immunomodulatory molecules making them potentially useful for constructing vaccines or therapeutics. For instance, the capsular polysaccharide A (PSA) of *Bacteroides fragilis* can prevent various inflammatory disorders and are being modified, conjugated and incorporated into nanoparticles which have exhibited immunological activity, demonstrating a chemical biology strategy for developing immunomodulatory nanomaterials from commensal microorganisms that has a potential use for vaccine and immunotherapeutic [74,154].

Manipulation of commensal bacteria for tolerogenic vaccines involves genetically engineering these naturally occurring microbes to deliver antigens in a way that promotes immune tolerance rather than immune activation [155]. These vaccines are intended to teach the immune system to recognize and tolerate specific antigens, thereby reducing or preventing harmful immune reactions, such as those seen in autoimmune diseases, allergies, or transplant rejection. Instead of activating the immune system to attack a target, tolerogenic vaccines promote the development of regulatory T cells (Tregs) and other mechanisms that suppress immune activity against specific antigens, aiming to prevent inflammation and tissue damage. Since commensal bacteria, such as *Lactobacillus* and *Bifidobacterium*, are already part of the body's microbiome and generally induce non-inflammatory responses, they provide an ideal platform for tolerogenic vaccine development. By engineering these bacteria to express specific autoantigens or allergens, they can present these antigens in a manner that encourages the development of regulatory T cells (Tregs) and other tolerance-promoting mechanisms. These responses reduce harmful immune reactions, such as in autoimmune diseases or allergies, by shifting the immune system away from a pro-inflammatory response toward immune regulation. This approach leverages the natural interaction between the immune system and the microbiome, using commensals as a safe and effective means to modulate immune tolerance and prevent immune-mediated diseases.

### 3.8 Integration of bacteria with nanoparticle-coating and microencapsulation

Aside from the genetic modifications done to attenuate bacteria for safe vaccine delivery or to enhance the antigen expression system for efficient induction of immune responses, other emerging advanced technologies have been recently integrated with the traditional bacterial vaccines such as coating live bacterial cells with synthetic nanoparticles and microencapsulation of bacteria (Fig. 4). The integration of nanoparticles with bacterial vaccines has shown enhancement in antigen delivery by encapsulating traditional bacterial vaccine antigens with nanoparticles, protecting them from degradation and enhancing their delivery to antigen-presenting cells (APCs). For example, although attenuated bacterial vaccines have been successfully utilized as carriers of oral delivery of vaccines, the oral infection of some bacterial strains is often low due to harsh acidic environment conditions in the stomach. An attempt to coat *Salmonella* with nanoparticles self-assembled from cationic polymers and plasmid DNA was demonstrated in the study by Hu et al., 2015. The protective nanoparticle coating layer could facilitate the effective escape of bacteria from phagosomes, enhance the acid tolerance of bacteria in the gastrointestinal tract and promote systemic circulation after oral inoculation [156].

Another strategy in the attempt to prolong the induction of protective immunity by an attenuated bacterial vaccine was demonstrated using alginate encapsulation which is frequently used to increase the persistence of probiotics [157]. Alginate encapsulation was done in the highly attenuated *Francisella tularensis* Live Vaccine Strain (LVS) and vaccination with the encapsulated strain showed increased survival in complement-active serum and increased antibody-mediated immunity [157]. Although there is an improved antibody-mediated immune response by the alginate-encapsulated bacteria, the induction of essential cell-mediated immune response appeared to be dampened which can be further improved possibly by the addition of conjugates that can lead to stimulation of cell-mediated immune response [157].

Biomimetic nanotechnology combines the principles of biology and nanotechnology to create systems that have biological processes, structures and functions that can be adapted in designing efficient and effective strategies to solve challenges in the medical field. Recently, nanotechnology has been employed to increase the potency of vaccine formulations through several advantages that nanoparticles offer, including the ability to protect the biological functions of encapsulated payloads, unified delivery of antigen and adjuvant and targeted cell delivery [158]. With these advantages, the integration of the concepts of nanotechnology with bacterial vaccines combines the strengths of both approaches to improve the efficacy, safety, and versatility of immunizations.

## 4 Challenges and limitations

Despite the tremendous potential demonstrated by trial experiments, translating bacteria-mediated immunization strategies into human applications remains a challenging task. Key challenges that must be addressed in bacteria-based formulations include issues with consistency, ineffective formulations and associated techniques, the need for extensive experimentation that can increase early development costs, stability issues and the potential for genetic alterations in bacteria species over time. A well-known example of a consistency-related issue is Coley's toxins, used in cancer treatment [159]. This formulation, consisting of a mixture of killed *Serratia marcescens* and *Streptococcus pyogenes* bacteria to stimulate the immune system extended the lives of some terminally ill patients by over a decade [160]. However, a lack of standardized protocols for reformulation led to inconsistencies in clinical trials, raising concerns about its reliability for human application [160].

In some cases, mutant bacterial strains lack sufficient potency to achieve the intended therapeutic outcome. For example, *Salmonella* Typhimurium VNP20009, a genetically modified strain developed for cancer therapy, has demonstrated some limitations in targeting and exploiting the tumor microenvironment [161]. Similarly, *Lactobacillus* GG, tested for treating inflammatory bowel disease, failed to produce consistent and robust therapeutic outcomes compared to standard treatments for ulcerative colitis [162]. Additionally, a bacterial vaccine developed to display Human papillomavirus (HPV) antigens failed to elicit a safe immune response [163] and faced challenges progressing through clinical trials. These examples collectively indicate that bacteria-mediated treatments in humans still face significant challenges regarding safety and efficacy, warranting further, in-depth investigation.

While bacterial vaccines offer numerous advantages in experimental settings, their development is accompanied by various challenges and limitations that require rigorous evaluation. These challenges can significantly impact vaccine efficacy, safety, and scalability, hindering their widespread adoption and effectiveness in preventing various infectious diseases. For example, variability in vaccine efficacy observed among different bacterial species and strains poses a substantial challenge. The BCG vaccine, the only licensed vaccine for TB, exemplifies this issue, as it demonstrates variable efficacy in protecting against severe forms of TB, particularly in children [164]. Similarly, the pertussis vaccine has shown varying effectiveness against *Bordetella pertussis* due to changes in circulating strains and waning immunity, highlighting the necessity for continuous adaptation of formulations to address evolving pathogens [165].

Another challenge lies in the limited immunogenicity of certain bacterial antigens, which necessitates the inclusion of adjuvants or multiple antigens in vaccine formulations to enhance or maintain intended immune responses. Additionally, concerns about undesirable immune reactions can raise safety issues. For instance, whole-cell pertussis vaccines have been associated with adverse events, prompting a shift toward acellular pertussis vaccines with improved safety profiles. Live attenuated bacterial vaccines also carry the risk of reverting to virulence or shedding in immunocompromised individuals, which requires careful evaluation of their safety.

Scalability and production challenges further limit the development and deployment of bacterial vaccines. Manufacturing processes often require specialized facilities and expertise, which can increase production costs and complicate logistics. Furthermore, the stability and shelf-life of bacterial vaccines can vary, necessitating strict storage and transportation conditions to maintain efficacy. Despite these obstacles, ongoing research is focused on overcoming these limitations and enhancing the effectiveness of bacterial vaccines. Innovations such as vector-based vaccines and DNA vaccines offer alternative strategies to overcome immunogenicity and scalability challenges [166]. Advances in adjuvant development and formulation technologies are also aimed at improving vaccine immunogenicity while minimizing adverse reactions [167].

Several concerns are also being raised when considering different routes of administration of this bacterial-based vaccine delivery. In some cases, especially with oral or nasal vaccine delivery, attenuated bacteria could cross mucosal barriers and enter the bloodstream, causing bacteremia or sepsis, especially in immunocompromised individuals [168]. There can also be a risk that the immune system might tolerate the antigens instead of mounting a robust immune response in orally or nasally administered bacteria [169]. Conversely, excessive stimulation of mucosal immunity could lead to mucosal inflammation or autoimmune responses, particularly in individuals predisposed to inflammatory conditions such as inflammatory bowel disease (IBD) [170] or asthma [171]. The different routes of administration may also result in varying levels of immune response. For example, orally administered vaccines may face challenges due to gastric acid and digestive enzymes that may degrade the bacterial strain or its antigens before reaching the gut-associated lymphoid tissue (GALT) [172]. On the other hand, nasal vaccines might result in lower systemic immunity since it is focused primarily on local mucosal immunity. In cases of intradermal or intramuscular routes, although they generally induce stronger systemic immune responses, they are less effective at generating mucosal immunity, which is crucial in combating pathogens that enter via mucosal surfaces. Administration of bacterial strains via oral or nasal routes could disrupt the natural microbiome causing dysbiosis, potentially leading to gastrointestinal disorders, increased susceptibility to infections, or reduced effectiveness of the local immune response [173].

Evaluation of bacterial vaccine strains in various animal models presents challenges, as differences in the infectivity of the bacterial strain may exist among different hosts. The development of safe and effective bacterial vaccine carriers for humans may be hindered by discrepancies observed in animal systems, where outcomes may not precisely reflect human responses. For instance, while *S*. Typhimurium may infect mice, it may not have similar effects on humans, and vice versa for *S.* Typhi [174]. Therefore, selecting appropriate animal models is critical for investigating bacterial vaccine efficacy and safety. Furthermore, although experimental studies on bacterial vaccine vectors have been conducted for decades, there are currently no commercial live recombinant bacterial vectors for human or veterinary use, aside from some progress in clinical studies with attenuated *Salmonella* Typhimurium and Typhi, *Vibrio cholera*, and *Listeria monocytogenes*. Concerns regarding bacterial virulence and uncontrolled immune responses also pose challenges to clinical trials and applications of bacterial vectors, highlighting the importance of addressing safety issues for individual vaccination and environmental considerations.

In summary, while bacterial vaccines offer considerable promise in preventing infectious diseases, they face challenges related to efficacy, safety, and scalability. Addressing these challenges requires a multidisciplinary approach involving ongoing research, innovation, and collaboration to develop safer, more effective, and globally accessible bacterial vaccines.

## 5 Emerging trends in utilizing bacteria for personalized vaccine development

Personalized bacterial vaccines represent an innovative approach to vaccine development that aims to tailor immunization strategies to individual patients based on their unique immune profiles and disease susceptibilities [158,175]. This concept capitalizes on advances in sequencing technologies and immunogenomics, allowing for a deeper understanding of the genetic makeup of pathogens and the host immune response. The process begins with the identification of specific antigens associated with a particular disease or pathogen, which can be determined through genomic sequencing and analysis. Researchers then select antigens that are highly immunogenic and likely to elicit a robust immune response. Once the antigens are identified, the next step involves designing a vaccine formulation that incorporates these antigens in a personalized manner. This may include selecting the most appropriate bacterial vector or carrier system to deliver the antigens, optimizing vaccine delivery routes and dosing schedules, and considering any potential adjuvants or immune modulators to enhance vaccine efficacy.

One of the primary advantages of personalized bacterial vaccines is their potential to overcome the limitations of traditional one-size-fits-all vaccines. By targeting specific antigens relevant to an individual's immune status and disease susceptibility, these engineered vaccines have the potential to generate more potent and durable immune responses. Additionally, personalized vaccines may minimize the risk of adverse reactions or vaccine-related complications by avoiding unnecessary exposure to antigens that the individual's immune system may not respond to effectively. Despite the promising potential of personalized bacterial vaccines, several challenges remain in their development and implementation, including regulatory hurdles, manufacturing complexities, and cost considerations. However, ongoing research efforts are focused on addressing these challenges to bring personalized bacterial vaccines closer to clinical reality.

**Fig. 6:**
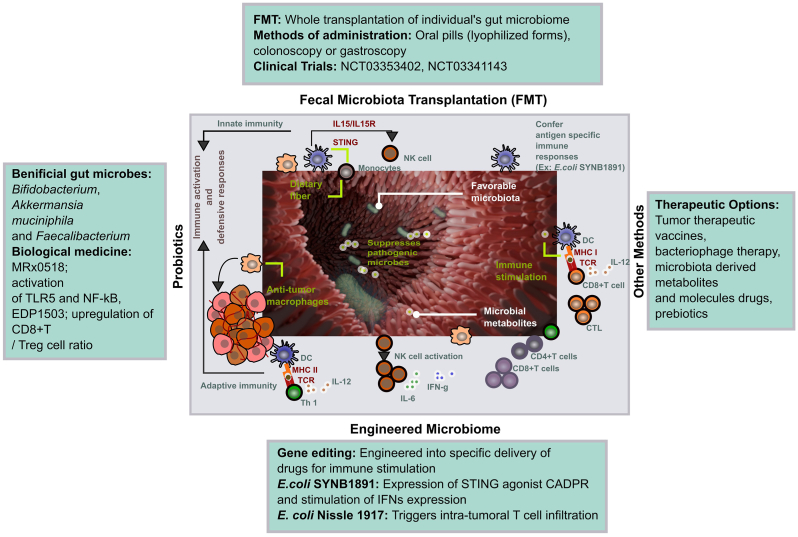
Microbiome therapy and personalized medicine. The dimensions of current microbiome therapeutic perspectives are demonstrated. Fecal microbiota transplantation (FMT), tumor therapeutics and microbial vaccine development, engineered microbiome, and probiotics and prebiotics applications are presented. Therapeutic bacterial strains, such as *E. coli* SYNB1891, *E. coli* Nissle 1917 are already applied for specific applications such as STING agonization and trigger intra-tumoral T cell responses respectively. Collective immune system stimulation by bacteria and their metabolites triggers the innate and adaptive immune responses and suppresses the growth of pathogenic bacterial species.

Microbiome-based immunotherapy is also an emerging field at the intersection of microbiology and immunology, where its principle is based on the intricate relationship between the human microbiome and the immune system. By understanding how the microbiome influences immune function and overall health, researchers are exploring innovative approaches to modulate the microbiome and restore immune homeostasis in various diseases. These approaches include fecal microbiota transplantation (FMT) to restore microbial balance [176,177], microbiome modulation through dietary interventions or microbial therapies [178], synthetic microbiome engineering to design tailored microbial communities, and the development of microbiome-targeted vaccines and immunomodulators [179] (Fig. 6). Recent advances in high-throughput sequencing technologies and computational tools have enabled deeper insights into the composition and function of the microbiome, facilitating the identification of microbial signatures associated with health and disease. Moreover, the advent of precision medicine has created new avenues for personalized microbiome-based interventions, where individualized treatment strategies can be designed based on an individual's microbiome profile. While still in its early stages, microbiome-based immunotherapy holds promise for revolutionizing disease treatment by harnessing the therapeutic potential of the human microbiome to prevent, manage, or even cure a range of conditions, from infectious diseases to autoimmune disorders and cancer.

## 6 Conclusion

Bacterial vaccines hold immense promise, with several emerging trends and future directions poised to revolutionize vaccine development and immunization strategies. One significant area of advancement lies in the development of next-generation bacterial vaccines. Innovations in molecular biology and vaccine technology are driving the creation of vaccines with enhanced efficacy, safety, and scalability. Novel platforms such as recombinant bacterial vectors, DNA vaccines, and RNA vaccines offer exciting opportunities for targeted antigen delivery and robust immune stimulation. Additionally, synthetic biology approaches enable the rational design of attenuated bacterial strains tailored to specific pathogens and populations, paving the way for highly effective vaccines against a range of infectious diseases. Integration of omics technologies represents another frontier in vaccine design. Genomics, transcriptomics, proteomics, and metabolomics enable comprehensive analyses of bacterial pathogens, host-pathogen interactions, and immune responses. Using these omics data, researchers can identify vaccine candidates, antigenic targets, and biomarkers of vaccine efficacy, guiding the development of vaccines with enhanced immunogenicity and effectiveness.

The intricate immunological inter-relationship of bacteria (microbiome) and the host enables novel avenues of advanced personal therapies such as fecal microbiota transplantation (FMT) and personalized therapies based on synthetic biology approaches. Furthermore, personalized and precision vaccinology approaches are transforming vaccine development strategies. Precision vaccinology aims to optimize vaccine responses by identifying predictive biomarkers of vaccine responsiveness, optimizing vaccine formulations, and tailoring vaccination schedules based on individual immune status. These personalized approaches have the potential to enhance vaccine effectiveness, minimize adverse reactions, and improve vaccine coverage and compliance, ultimately advancing preventive medicine. Advancing the bacterial engineering approaches and strengthening interdisciplinary collaborations will help overcome existing challenges and pave the way for transformative breakthroughs in preventive medicine. By harnessing the power of science and technology, the prospect of achieving global health equity becomes more feasible.

## CRediT authorship contribution statement

**Khristine Kaith S. Lloren:** Writing – review & editing, Writing – original draft, Visualization, Formal analysis, Data curation, Conceptualization. **Amal Senevirathne:** Writing – review & editing, Writing – original draft, Visualization, Formal analysis, Data curation, Conceptualization. **John Hwa Lee:** Writing – review & editing, Supervision, Funding acquisition, Conceptualization.

## Declaration of competing interest

The authors declare that they have no known competing financial interests or personal relationships that could have appeared to influence the work reported in this paper.

## Data Availability

No data was used for the research described in the article.
